# Impact of Systemic Volume Status on Cardiac Magnetic Resonance T1 Mapping

**DOI:** 10.1038/s41598-018-23868-4

**Published:** 2018-04-03

**Authors:** Marlies Antlanger, Stefan Aschauer, Andreas A. Kammerlander, Franz Duca, Marcus D. Säemann, Diana Bonderman, Julia Mascherbauer

**Affiliations:** 1 Department of Internal Medicine III, Division of Nephrology and Dialysis, Medical University of Vienna, Vienna, Austria; 2 Department of Internal Medicine II, Division of Cardiology, Medical University of Vienna, Vienna, Austria; 3 6th Department of Internal Medicine, Nephrology and Dialysis, Wilhelminenspital, Vienna, Austria; 4 Sigmund Freud Private University, Medical School, Vienna, Austria

## Abstract

Diffuse myocardial fibrosis is a key pathophysiologic feature in heart failure and can be quantified by cardiac magnetic resonance (CMR) T1 mapping. However, increases in myocardial free water also prolong native T1 times and may impact fibrosis quantification. Thus far, the impact of systemic patient volume status remains unclear. In this study, native T1 time by CMR was investigated in hemodialysis (HD) patients (n = 37) and compared with healthy controls (n = 35). Volume status was quantified by bioimpedance spectroscopy and correlated with CMR T1 time. While no differences between HD patients and controls were present with regard to age (p = 0.180), height (p = 0.535), weight (p = 0.559) and left ventricular (LV) ejection fraction (p = 0.273), cardiac size was significantly larger in HD patients (LV end-diastolic volume 164 ± 53 vs. 132 ± 26 ml, p = 0.002). Fluid overloaded HD patients had significantly longer native T1 times than normovolemic HD patients and healthy controls (1,042 ± 46 vs. 1,005 ± 49 vs. 998 ± 47 ms, p = 0.030). By regression analysis, T1 time was significantly associated with fluid status (r = 0.530, p = 0.009, post-HD fluid status). Our data strongly indicate that native CMR T1 time is significantly influenced by systemic volume status. As fluid overload is common in patients with cardiovascular diseases, this finding is important and requires further study.

## Introduction

Cardiac magnetic resonance imaging (CMR) including T1 mapping is increasingly used to characterize myocardial disease^1,2^. Native T1 values are a composite signal from myocytes and extracellular volume (ECV). The two most important determinants of an increase in native T1 are edema and fibrosis or amyloid. Native T1 time has been studied as a surrogate marker of diffuse myocardial fibrosis in heart failure with preserved and reduced ejection fraction (HFpEF and HFrEF)^3,4^, and also in myocardial inflammation and subsequent edema^5,6^. Especially in heart failure, fluid overload is a frequently encountered clinical problem. However, it currently remains unclear to what extent systemic fluid overload influences CMR T1 times and whether a differentiation between fibrosis and overhydration is possible in affected patients. Patients with end-stage renal disease on maintenance hemodialysis (HD), who are closely followed with regard to their fluid status, frequently develop left ventricular hypertrophy and diastolic dysfunction^7^. This has been linked to a high prevalence of risk factors such as hypertension, coronary artery disease, chronic inflammation and diabetes^8^. Chronic kidney disease patients frequently develop left ventricular hypertrophy as well as diastolic dysfunction with the most extreme forms typically found in dialysis patients^9^. The combined effects of pre-existing comorbidities, the continuous strain put on the myocardium through hemodialysis and ultrafiltration and the effects of chronic fluid overload, which remain even after ultrafiltration, lead to a particularly high HFpEF prevalence in HD patients^10^.

Recently, CMR was applied as a novel tool in HD patients and significantly prolonged native T1 values were found^11,12^. In an intricate piece of work, Buchanan *et al*. were able to show stable T1 times by repeated intradialytic CMR during a HD session, suggesting unchanged myocardial water content during the ultrafiltration process^13^. Yet, fluid status was not specified and it was acknowledged that additional studies are needed to clarify this issue. Recently, bioimpedance methods, which objectively and quantitatively measure patients’ volume status have been implemented in the care of HD patients and were also used to measure volume status in heart failure patients^14,15^. Additionally, it remains currently unclear whether other morphological myocardial changes found in HD patients exert an influence on the measurement of native T1 time.

In the present study, the potential association of volume status and native T1 time by CMR was investigated in consecutive HD patients and compared with results from healthy controls.

## Methods

### Study participants

37 patients undergoing maintenance HD at the Medical University of Vienna were consecutively enrolled. Medical history, information on residual urinary output, and dialysis-associated parameters were collected at study entry. All patients underwent a comprehensive echocardiographic evaluation by board certified cardiologists using scanners such as GE Vivid 7 and Vivid S70 (GE Healthcare, Wauwatosa, WI). History of coronary artery disease was defined as prior need for revascularization (percutaneous coronary intervention or coronary artery bypass surgery). Patients diagnosed with HFrEF according to the 2016 guidelines of the European Society of Cardiology (defined by a left ventricular ejection fraction <40% on echocardiography) were excluded^16^. Further exclusion criteria were defined as dialysis treatment <3 months, prior heart or lung transplantation, known significant untreated coronary artery or significant aortic/mitral valve disease, congenital heart disease, and chronic obstructive pulmonary disease GOLD IV.

All participants gave written informed consent. The study was approved by the ethics committee of the Medical University of Vienna (EC#1036/2013) and performed according to the Declaration of Helsinki. Native T1 reference values were derived from 35 healthy controls. The datasets generated during and/or analyzed during the current study are available from the corresponding author on reasonable request.

### Cardiac magnetic resonance imaging

**Figure 1:**
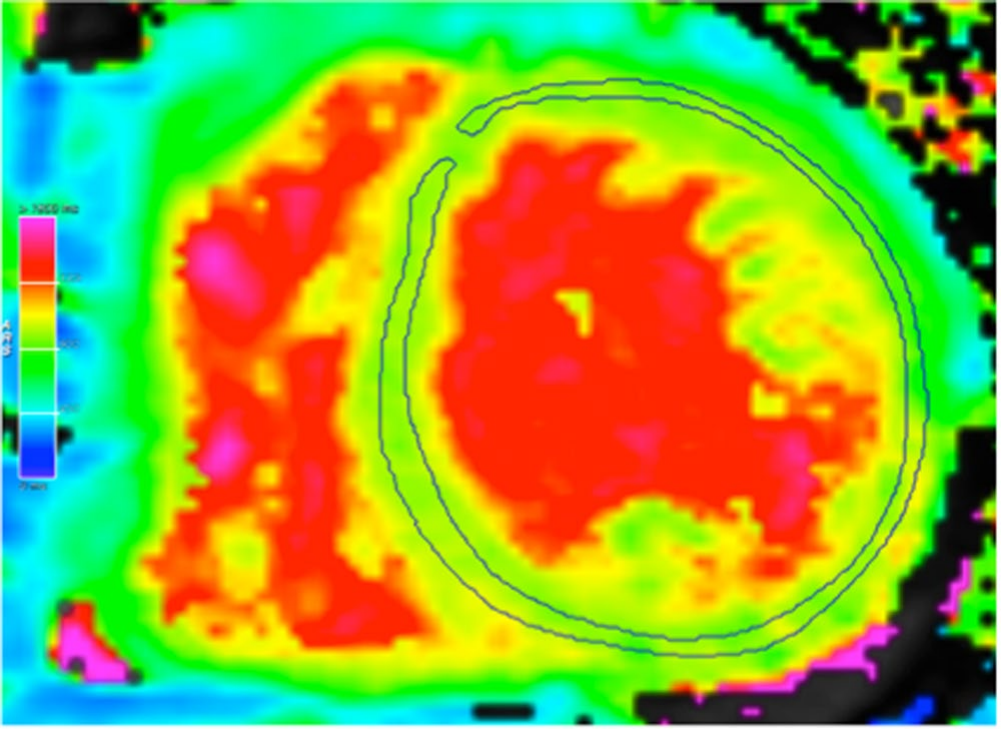
Native T1 map of a HD patient. Midventricular short-axis slice, myocardial region of interest was traced manually (circled area). Modified Look-Locker inversion recovery sequence with a 5(3)3 prototype was used to calculate T1 time.

All CMR studies were performed by board-certified physicians on a 1.5 Tesla cardiac-dedicated clinical magnetic resonance system (MAGNETOM Avanto, Siemens Medical Solutions, Erlangen, Germany) post-dialysis or on an interdialytic day. The CMR protocol consisted of a functional study without late gadolinium enhancement imaging. 3 standard long-axis slices and a stack of contiguous short-axis slices (slice thickness: 10 mm, no gap, 30 phases/RR-interval) were acquired with electrocardiography-gated steady-state free-precession cine-images (repetition time 2.9 ms, echo time 1.2 ms, flip angle 80°, matrix 256 × 146, field of view typically 340 mm, bandwidth 930 Hz/pixel) in breath-hold technique. For quantification of left and right ventricular ejection fraction and chamber volumes, the endocardial and epicardial contours were traced manually in end-systole and -diastole using dedicated software. The left ventricular (LV) end-diastolic volume was indexed to height in cm. Interventricular septum thickness was measured in the 4-chamber view. For native T1 mapping, a modified Look-Locker inversion recovery (MOLLI) with a 5(3)3 prototype was used. This technique allows an inline, pixel-by-pixel based T1-map within one breath-hold. Motion correction was used for all series of images acquired. Parameters for T1 sequences were: inversion time 120 ms with an increment of 80 ms, measured matrix size 256 × 144, reconstructed matrix size 256 × 218, phase encoding resolution 66%, and field of view 85%. T1 maps were acquired in a mid-ventricular short axis as well as a 4-chamber view. No systematic base-to-apex native T1 variation was found. Left ventricular myocardium was defined as region of interest with a clear distance from the endomyocardial border to avoid blood pool T1 signals (Fig. 1).

### Fluid status assessment

All HD patients underwent pre-dialysis bioimpedance spectroscopy assessment to objectively determine and quantify their volume status and body composition. For this purpose, the body composition monitor (BCM, Fresenius Medical Care, Germany) was used. Based on a fluid model using body compartment resistance, extracellular water, intracellular water and total body water as well as adipose and lean tissue mass were measured^14,17^.

Extracellular fluid overload was then determined through a BCM algorithm. First, normohydration status was calculated, i.e. the expected normal value for extracellular volume for the patient’s weight. Subsequently, the tissue hydration status was determined as the difference between the actual extracellular volume and the ideal normohydration volume. Fluid overload was calculated as an absolute value in liters and in percent above the normal extracellular volume. In previous studies, the cut-off threshold for pre-HD fluid overload was defined as >15% relative to the extracellular volume, largely corresponding to an absolute value of 2.5 liters^18^. All patients classified as fluid overloaded had both a percental expansion of the extracellular volume above 15% as well as an absolute expansion above 2.5 liters.

Further, ultrafiltration volume was included in the measurement to calculate pre- and post-dialysis fluid overload. Post-HD hypervolemia was defined as volume expansion >0% extracellular volume.

### Statistical analysis

Demographic data are presented as mean values with standard deviation (SD) if normally distributed. Paired Student’s *t*-test was performed to determine differences between groups. Non-normally distributed values are described as medians [interquartile range] and Mann-Whitney-U-test was applied. Categorical data were assessed using the χ^2^ test. Group-wise comparison of T1 time was carried out with ANOVA. Correlation calculations were performed using Pearson’s correlation coefficient. A multiple regression analysis was run to predict native T1 time from factors, which were suspected to affect native T1 time (N-terminal prohormone of brain natriuretic peptide (NT pro-BNP), fluid overload, LV end-diastolic volume and LV mass). NT pro-BNP was logarithmized for multiple regression analysis. All statistical analyses were conducted using the software packages SPSS System for Mac version 22.0.0 (SPSS, Inc., 2010, Chicago, IL).

## Results

### Baseline characteristics

Table 1 shows baseline demographic and fluid status data of HD patients. In general, HD patients had enlarged hearts with a mean end-diastolic LV diameter of 48 ± 7 mm and a mean left atrial (LA) diameter of 56 ± 8 mm. The mean estimated systolic pulmonary artery pressure was also elevated (46 ± 12 mmHg). Almost all HD patients showed signs of diastolic dysfunction.

**Table 1:** Baseline characteristics.

Variable	HD patients (n = 37)
**HD parameters**	
HD vintage, years	3.0 ± 4.6
Prior KTX, %	43
Residual urine >500 ml/d, %	48
AVF, %	78
Ultrafiltration, L	2.3 ± 1.3
HD duration, h	4.0 ± 0.3
Blood flow, ml/min	293 ± 45
RR_sys_, mmHg	146 ± 23
RR_diast_, mmHg	80 ± 15
**Comorbidities**	
Underlying renal disease, %	
Vascular	8
Glomerular	31
Polycystic	8
Tubulointerstitial	8
Diabetic	11
Other/unknown	33
Arterial hypertension, %	87
Coronary artery disease, %	27
Atrial fibrillation, %	8
Diabetes mellitus, %	14
COPD, %	14
Peripheral artery disease, %	14
Cerebrovascular disease, %	8
**Laboratory parameters**	
Hemoglobin, g/dL	10.4 ± 1.0
Ferritin, μg/l	318 ± 335
Transferrin saturation, %	20 ± 12
PTH, pg/mL	459 ± 474
25-OH vitamin D, nmol/L	45 ± 30
1,25-OH vitamin D, pg/mL	20 ± 13
HbA_1_c, %	5.3 ± 1.1
Triglycerides, mg/dL	157 ± 98
Total cholesterol, mg/dL	166 ± 40
Low-density cholesterol, mg/dL	94 ± 32
High-density cholesterol, mg/dL	43 ± 19
NT pro-BNP, pg/mL	3,889 [2,012-12,468]
**Echocardiography parameters**	
Left ventricular diameter, mm	48 ± 7
Right ventricular diameter, mm	32 ± 6
Left atrial diameter, mm	56 ± 8
Right atrial diameter, mm	54 ± 7
Interventricular septum, mm	14.7 ± 2.8
sPAP, mmHg	46 ± 12
Peak E ms/s	0.9 ± 0.4
Peak A ms/s	0.8 ± 0.2
E/A ratio	0.56 ± 0.63
E’ medial m/s	0.07 ± 0.02
E/E’ ratio	13.5 ± 7.6
**Diastolic dysfunction**	
None, %	6
Grade 1, %	61
Grade 2, %	24
Grade 4, %	9
**Bioimpedance parameters**	
Fluid overload pre-HD, L	2.6 ± 2.3
Fluid overload pre-HD, %ECV	13.9 ± 12.0
Fluid overload post-HD, L	0.4 ± 2.7
Fluid overload post-HD, %ECV	1.8 ± 15.7
Total body water, L	38.2 ± 7.3
Extracellular volume, L	18.2 ± 3.1
Intracellular volume, L	19.8 ± 4.6
Lean tissue mass, kg	42.7 ± 12.6
Adipose tissue mass, kg	25.6 ± 12.0
Lean tissue index	14.6 ± 3.4
Fat tissue index	9.2 ± 4.6
Body cell mass, kg	24.2 ± 8.5

HD, hemodialysis; KTX, kidney transplantation; AVF, arteriovenous fistula; RR, blood pressure; COPD, chronic obstructive pulmonary disease; PTH, parathyroid hormone; NT pro-BNP, N-terminal prohormone of brain natriuretic peptide; sPAP; systolic pulmonary artery pressure; E, early medial diastolic mitral velocity; A, late medial mitral velocity; E’, early lateral diastolic mitral velocity; ECV, extracellular volume.

On average, a pre-HD fluid overload of 2.6 ± 2.3 L above the ideal extracellular volume was found, corresponding to a relative value of 13.9 ± 12.0% above ideal extracellular volume. Overall, 43% of all HD patients were fluid overloaded.

### Cardiac magnetic resonance imaging

Table 2 shows CMR imaging results of HD patients in comparison with healthy controls. No differences were found with regard to age, height and weight. However, LV hypertrophy was more pronounced in HD patients with a median [IQR] interventricular septal thickness of 14 [11–16] compared to 9 [8–10] mm in the control group (p < 0.001); LV myocardial mass was 172 ± 53 versus 108 ± 21 g in controls (p < 0.001). While left and right ventricular (RV) ejection fractions (EF) were comparable (LV-EF 63 ± 9 vs. 65 ± 7%, p = 0.273; RV-EF 59 ± 7 vs. 60 ± 8%, p = 0.581), LA and LV size were significantly increased in HD patients (LA diameter 60 ± 9 vs. 54 ± 6 mm, p = 0.002; LV end-diastolic diameter 51 ± 8 vs. 47 ± 5 mm, p = 0.011). Cardiac output was higher in HD patients (7.3 ± 2.2 vs. 5.9 ± 1.7 L/min, p = 0.004). The diameter of the pulmonary artery was significantly wider in HD patients, indicating higher pressures in the pulmonary vascular bed^19,20^.

**Table 2:** Cardiac magnetic resonance imaging variables.

Variable	Healthy controls (n = 35)	HD patients (n = 37)	p-value
Age, years	43 ± 17	49 ± 16	0.180
Height, cm	170 ± 10	169 ± 9	0.535
Weight, kg	71 ± 12	70 ± 11	0.559
BMI, kg/m^2^	24.5 ± 3.6	24.4 ± 3.4	0.873
IVS, mm	9 [8–10]	14 [11–16]	**<0.001**
LV-EDD, mm	47 ± 5	51 ± 8	**0.011**
RV-EDD, mm	37 ± 6	37 ± 6	0.961
LA, mm	54 ± 6	60 ± 9	**0.002**
LA area, cm^2^	21 ± 5	27 ± 8	**0.001**
RA, mm	55 ± 6	57 ± 9	0.233
RA area, cm^2^	21 [18–24]	23 [21–26]	0.731
Ascending aorta, mm	32 ± 4	35 ± 4	**0.005**
Pulmonary trunk, mm	22 ± 3	29 ± 4	**<0.001**
LV-EF, %	65 ± 7	63 ± 9	0.273
LV-EDV, mL	132 ± 26	164 ± 53	**0.002**
LV-ESV, mL	45 [37–54]	53 [44–74]	**0.014**
LV-SV, mL	85 ± 19	102 ± 32	**0.009**
RV-EF, %	60 ± 8	59 ± 7	0.581
RV-EDV, mL	139 ± 27	160 ± 45	**0.017**
RV-ESV, mL	57 ± 16	66 ± 22	**0.040**
RV-SV, mL	82 ± 19	95 ± 28	**0.032**
Cardiac output, L/min	5.9 ± 1.7	7.3 ± 2.2	**0.004**
LV mass, g	108 ± 21	172 ± 53	**<0.001**
Native T1 time (SA), ms	998 ± 47	1,022 ± 50	**0.043**

HD, hemodialysis; BMI, body mass index; IVS, interventricular septum; LV, left ventricle, RV, right ventricle, RA, right atrium; EDD, enddiastolic diameter; LA, left atrium; EF, ejection fraction; EDV, end-diastolic volume; ESV, end-systolic volume; SV, stroke volume; SA, short axis.

Native myocardial T1 time was significantly longer in the HD group (1,022 ± 50 vs. 998 ± 47 ms, p = 0.043, suppl. Figure 1).

### Impact of fluid status on cardiac magnetic resonance imaging variables

Bioimpedance measurements (n = 30) were analyzed to discriminate between normohydrated (n = 17) and fluid overloaded (n = 13) HD patients (Table 3). Here, NT pro-BNP was found to be significantly higher in the hypervolemic group compared to the normovolemic group (p = 0.001). However, no significant correlation was found between NT pro-BNP and native T1 time (r = 0.223, p = 0.205). Furthermore, fluid overloaded patients had a significantly higher LV mass, lower body mass index and higher rates of coronary artery disease.

**Table 3:** Clinical and CMR values of fluid overloaded versus normovolemic HD patients.

Variable	Normohydrated (n = 17)	Fluid overloaded (n = 13)	p-value
Fluid overload pre-HD, %ECV	5.8 ± 8.5	24.5 ± 6.1	**<0.001**
Fluid overload post-HD, %ECV	−9.4 ± 12.3	13.9 ± 8.2	**<0.001**
Age, years	48 ± 19	53 ± 14	0.441
BMI, kg/m^2^	26.3 ± 3.4	22.8 ± 2.0	**0.003**
HD vintage, years	2.1 ± 1.9	5.0 ± 7.0	0.110
RR_sys_, mmHg	146 ± 24	148 ± 25	0.856
RR_diast_, mmHg	80 ± 16	80 ± 16	0.995
Hemoglobin, g/dL	10.5 ± 0.9	10.4 ± 1.1	0.819
NT pro-BNP, pg/mL	2,525 [1,493–3,908]	11,694 [5,350–20,424]	**0.001**
Coronary artery disease, %	18	54	**0.045**
Atrial fibrillation, %	6	15	0.397
Diabetes mellitus, %	6	23	0.204
IVS, mm	13 ± 3	14 ± 3	0.526
LA, mm	59 ± 7	61 ± 13	0.532
LV-EF, %	66 ± 6	62 ± 10	0.247
LV-EDV, mL	140 ± 41	186 ± 62	**0.021**
RA, mm	56 ± 6	60 ± 11	0.214
RV-EF, %	59 ± 8	59 ± 6	0.913
RV-EDV, mL	148 ± 36	173 ± 58	0.162
LV cardiac output, L/min	6.7 ± 1.6	7.9 ± 3.0	0.193
RV cardiac output, L/min	6.4 ± 1.3	7.0 ± 2.7	0.445
LV mass, g	147 ± 39	194 ± 64	**0.021**

HD, hemodialysis, ECV, extracellular volume; BMI, body mass index; RR, blood pressure; NT pro-BNP, N-terminal prohormone of brain natriuretic peptide; IVS, interventricular septum; LA, left atrium; LV, left ventricular; EF, ejection fraction; LV-EDV, left ventricular end-diastolic volume; RA, right atrium; RV, right ventricle.

With respect to CMR, a significantly longer native T1 time was detected among patients with fluid overload, while values of normohydrated patients were not different from values of healthy controls (1,042 ± 46 vs. 1,005 ± 49 vs. 998 ± 47 ms, p = 0.030, suppl. Figure 2).

**Figure 2:**
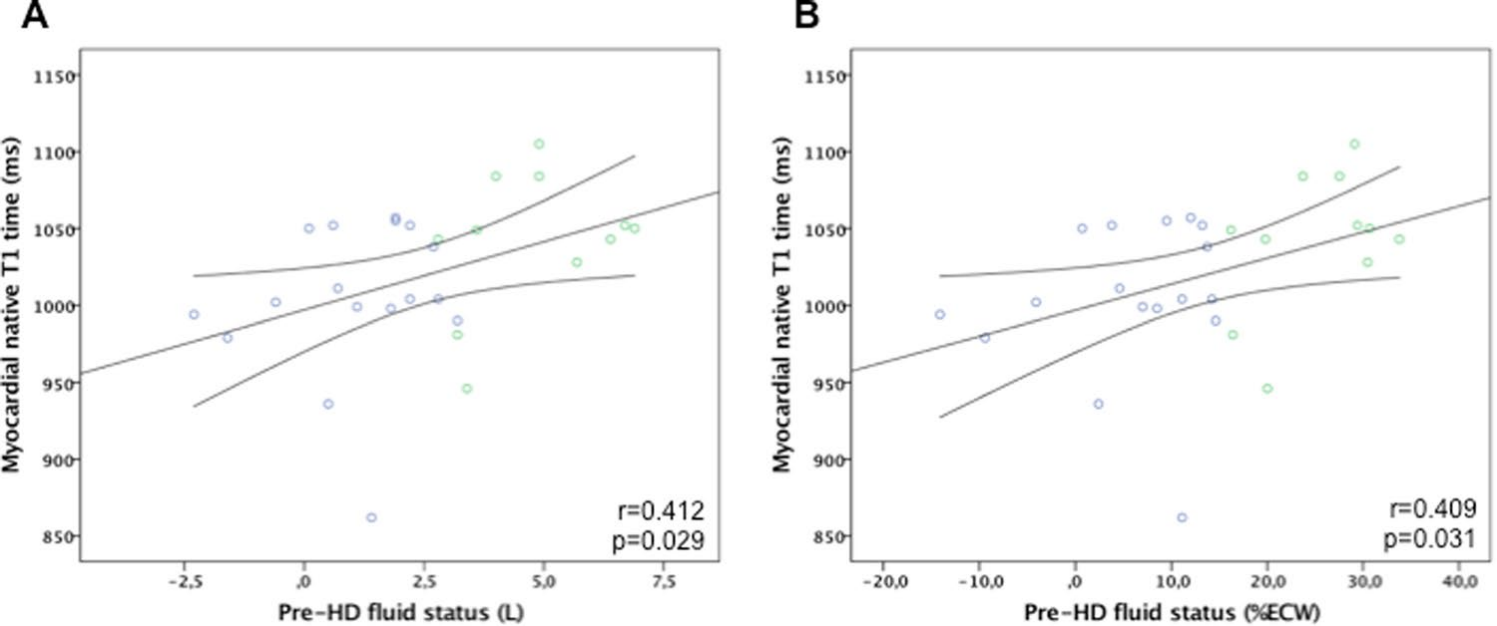
Correlation plots of fluid status and myocardial T1 time (short axis). (**A**) Absolute pre-HD fluid status in Liters above/below ideal extracellular volume. (**B**) Relative pre-HD fluid status in % above/below ideal extracellular volume. Blue circles represent normovolemic patients; green circles represent fluid overloaded patients.

**Figure 3:**
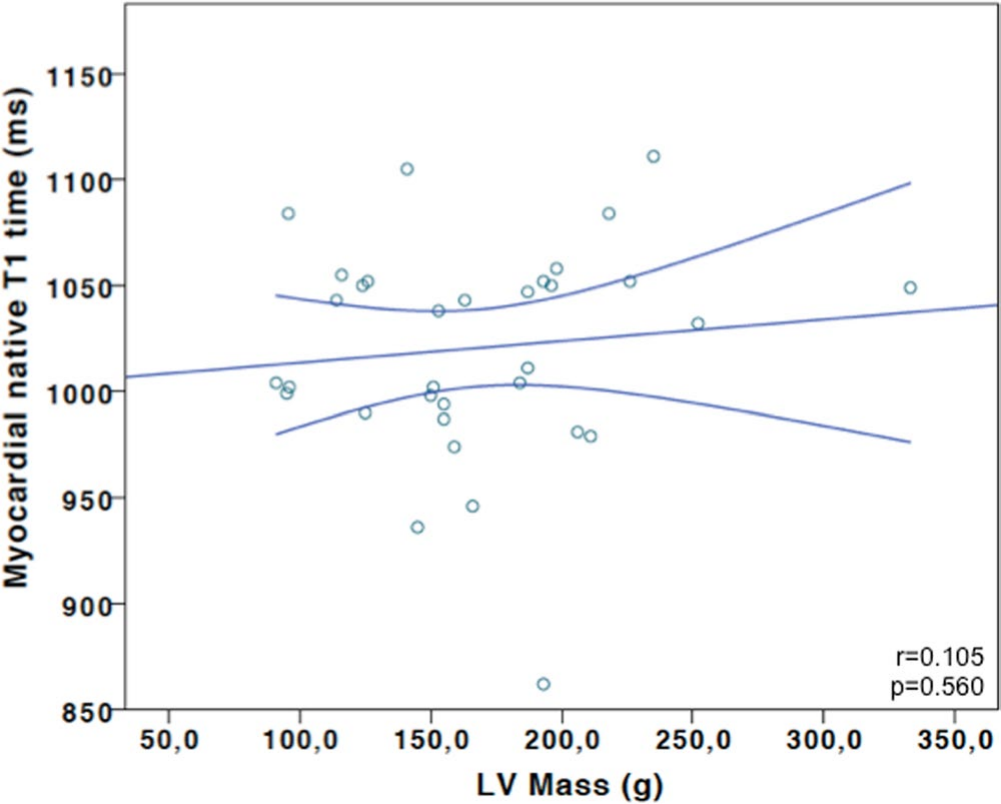
Correlation plot of left ventricular mass and myocardial T1 time (HD patients only).

By linear regression analysis, a significant correlation was found between fluid status and native T1 time (Fig. 2A,B). A similarly strong correlation was found between calculated post-HD fluid status and native T1 time (suppl. Figure 3) while LV mass was not significantly correlated with native T1 time (Fig. 3).

By multiple regression analysis including serum NT pro-BNP, LV end-diastolic volume and LV mass, fluid status was the only parameter that was significantly associated with myocardial T1 time (*F*(1,24) = 5,207, r = 0.422, p = 0.032).

## Discussion

The present study indicates that systemic fluid status impacts results of myocardial native T1 mapping by CMR in HD patients. This effect appears sufficiently large to represent a potential confounder for the interpretation of native T1 not only in HD patients but also patients with other conditions undergoing CMR.

Recently, T1 mapping has gained increasing importance in the diagnosis and prognostic evaluation of various myocardial pathologies^1,2^. Corresponding to an increase in extracellular volume, a linear prolongation of native T1 time has been observed^21^. Yet, high extracellular volume may be the result of various pathological processes: deposition of interstitial protein, such as in amyloidosis, leads to structural myocardial alterations and prolonged native T1 times^22,23^. Interstitial edema (secondary to myocardial infarction and/or myocarditis) causes an increase in extracellular volume^5,24^, and diffuse myocardial fibrosis in cardiomyopathy or heart failure can also increase native T1 times^2,3^.

However, the impact of systemic fluid status on native myocardial T1 time by CMR has not been addressed so far, although fluid overload is common both among patients suffering from renal failure and/or cardiovascular disease^25,26^. Lately, bioimpedance techniques stemming from HD research have become available to quickly and non-invasively determine a patient’s fluid status as well as other body composition parameters^14,27^. Patients with end-stage renal failure are usually in a highly controlled state of fluid gain and removal. In the present study patients on maintenance HD were invited to undergo CMR imaging including T1 mapping. The respective data were related to their fluid status and, in addition, compared with data from healthy controls.

Overall, cardiac size was significantly increased in HD patients compared to controls. This might potentially be due to the increased myocardial demand through long-standing arterial hypertension and vascular stiffening caused by mineral-bone disease and chronic inflammation^28–30^. Furthermore, it is known that cardiac output increases by 10–17% after the creation of an arterio-venous fistula^31,32^. Cases of high-output heart failure due to the overload caused by arterio-venous fistulas or grafts have previously been reported^33,34^.

Furthermore, those patients who proved to be fluid overloaded differed clinically from normohydrated HD patients: they had a significantly lower body mass index, higher NT pro-BNP serum levels and a higher rate of previously documented coronary artery disease, which are findings that have previously been reported in the literature^35,36^. Even though fluid overloaded HD patients had significantly lower body mass indices than normovolemic patients, their cardiac mass and size were larger. This stands in contrast with Pfaffenberger *et al*., who described that height and weight directly affect cardiac size^37^. Nevertheless, our finding fits in the “reverse epidemiology” concept of HD patients^36^, in which obesity is regarded a protective survival feature as well as the “obesity paradox” for HF patients^38^, where a survival advantage was proven for moderately obese patients.

Importantly, native myocardial T1 time was significantly longer in fluid overloaded HD patients, while the normohydrated group exhibited values comparable to healthy controls, suggesting an influence of hypervolemia on this measure. By multiple regression analysis, only fluid overload remained significantly associated with native T1 time, while other parameters, which are frequently related with myocardial disease in HD patients (i.e. LV-EDV, LV mass, NT pro-BNP) were not significantly correlated with T1 time on multiple regression analysis. In line with our results, Verbrugge and coworkers recently published their study on “Global myocardial oedema in advanced decompensated heart failure“ based on T2 measurements^39^. They showed that myocardial T2 times of congested heart failure patients significantly decreased after decongestion and under optimal heart failure treatment. The effect of treatment was monitored by invasive hemodynamics. Interestingly, psoas muscle T2 times also decreased after treatment, but not significantly. Furthermore, the correlation between quantitative myocardial T2 value change and net fluid balance was poor and non-significant. Thus, it is not clear from these data whether changes in T2 times were solely caused by an isolated loss of myocardial water or by improved cardiac function, as reflected by significantly higher mixed venous saturation and cardiac output after treatment. However, the current mainstay for the assessment of extracellular matrix expansion by CMR is the assessment of extracellular volume by T1 mapping. To our knowledge, fluid overload has so far not been related with T1 time prolongation.

Recently, Dekker *et al*. showed in a large patient cohort that pre-HD normovolemia as well as post-HD fluid depletion yield the best results with regard to overall survival^40^. It can therefore be concluded that keeping HD patients “dry” and even dialyzing them slightly below their dry weight is crucial with regard to long-term outcomes. In line with this report, fluid overload was shown to be associated with inferior event-free survival in HFpEF patients, who frequently suffer from additional renal impairment and systemic congestion^15^. Miller *et al*. determined total blood volume in these patients in a small pilot trial and found significantly elevated levels^41^. Closing the circle of these findings, it appears that diastolic dysfunction, which is frequently found in HD patients, is not only associated with interstitial fibrosis but also extracellular fluid volume expansion. As this condition seems to impact on CMR T1 time measurements, it requires careful evaluation.

Some limitations need to be discussed: the analyzed cohort was small and the results should therefore be interpreted with caution; however, so far this is the only study that systematically evaluated the association of fluid status objectively determined by bioimpedance measurements and native T1 time. Additionally, only 30 out of 37 bioimpedance measurements were technically sound and could be included in the analysis. This number was reached by excluding measurements with a data quality <90 (on a scale of 0–100 as displayed by the Cole-Cole plot derived device output) and measurements that did not yield any results at all through the bioimpedance device. Furthermore, fluid overload was accompanied by other patient-specific factors that distinguished normovolemic from hypervolemic patients. Nevertheless, on multiple regression analysis only fluid status remained significantly associated with native T1 time. T2 times were not analyzed in the present study. More interventional studies using bioimpedance-controlled dry weight reduction are needed to clarify to what extent systemic volume overload can influence CMR T1 times and the assessment of extracellular volume.

In conclusion, we demonstrate that native T1 time by CMR is influenced by patients’ systemic fluid status. Native T1 time was profoundly prolonged in fluid overloaded HD patients, while normohydrated HD patients had T1 times comparable to healthy controls. As fluid overload is common both in patients on dialysis as well as patients with myocardial pathologies, further studies assessing the impact of fluid status on CMR T1 mapping are urgently required.

## Electronic supplementary material

Supplementary Figures

## References

[1] Taylor AJ, Salerno M, Dharmakumar R, Jerosch-Herold M (2016). T1 Mapping: Basic Techniques and Clinical Applications. JACC Cardiovasc Imaging.

[2] Kammerlander AA (2016). T1 Mapping by CMR Imaging: From Histological Validation to Clinical Implication. JACC Cardiovasc Imaging.

[3] Puntmann VO (2013). Native T1 mapping in differentiation of normal myocardium from diffuse disease in hypertrophic and dilated cardiomyopathy. JACC Cardiovasc Imaging.

[4] Mascherbauer, J. *et al*. Cardiac Magnetic Resonance Post-Contrast T1 Time is Associated with Outcome in Patients with Heart Failure and Preserved Ejection Fraction. *Circ Cardiovasc Imaging*, 10.1161/CIRCIMAGING.113.000633 (2013). 10.1161/CIRCIMAGING.113.000633 24036385

[5] Ferreira VM (2012). Non-contrast T1-mapping detects acute myocardial edema with high diagnostic accuracy: a comparison to T2-weighted cardiovascular magnetic resonance. J Cardiovasc Magn Reson.

[6] Lurz P (2016). Comprehensive Cardiac Magnetic Resonance Imaging in Patients With Suspected Myocarditis: The MyoRacer-Trial. J Am Coll Cardiol.

[7] Wang AY (2013). Heart failure with preserved or reduced ejection fraction in patients treated with peritoneal dialysis. Am J Kidney Dis.

[8] Sarnak MJ (2003). Kidney disease as a risk factor for development of cardiovascular disease: a statement from the American Heart Association Councils on Kidney in Cardiovascular Disease, High Blood Pressure Research, Clinical Cardiology, and Epidemiology and Prevention. Circulation.

[9] Park M (2012). Associations between kidney function and subclinical cardiac abnormalities in CKD. J Am Soc Nephrol.

[10] Antlanger M (2017). Heart Failure with Preserved and Reduced Ejection Fraction in Hemodialysis Patients: Prevalence, Disease Prediction and Prognosis. Kidney Blood Press Res.

[11] Graham-Brown MP (2016). Novel cardiac nuclear magnetic resonance method for noninvasive assessment of myocardial fibrosis in hemodialysis patients. Kidney Int.

[12] Rutherford E (2016). Defining myocardial tissue abnormalities in end-stage renal failure with cardiac magnetic resonance imaging using native T1 mapping. Kidney Int.

[13] Buchanan, C. *et al*. Intradialytic Cardiac Magnetic Resonance Imaging to Assess Cardiovascular Responses in a Short-Term Trial of Hemodiafiltration and Hemodialysis. J *Am Soc Nephrol*, 10.1681/ASN.2016060686 (2016). 10.1681/ASN.2016060686 PMC5373461 28122851

[14] Chamney PW (2007). A whole-body model to distinguish excess fluid from the hydration of major body tissues. Am J Clin Nutr.

[15] Koell, B. *et al*. Fluid status and outcome in patients with heart failure and preserved ejection fraction. I*nt J Cardiol*, 10.1016/j.ijcard.2016.12.080 (2016). 10.1016/j.ijcard.2016.12.080 PMC6197425 28062131

[16] Ponikowski P (2016). 2016 ESC Guidelines for the Diagnosis and Treatment of Acute and Chronic Heart Failure. Rev Esp Cardiol (Engl Ed).

[17] Passauer J, Petrov H, Schleser A, Leicht J, Pucalka K (2010). Evaluation of clinical dry weight assessment in haemodialysis patients using bioimpedance spectroscopy: a cross-sectional study. Nephrol Dial Transplant.

[18] Wizemann V (2009). *T*he mortality risk of overhydration in haemodialysis patients. Nephrol Dial Transplant.

[19] Kammerlander AA (2017). Diameter of the Pulmonary Artery in Relation to the Ascending Aorta: Association with Cardiovascular Outcome. Radiology.

[20] Karakus G (2015). *P*ulmonary artery to aorta ratio for the detection of pulmonary hypertension: cardiovascular magnetic resonance and invasive hemodynamics in heart failure with preserved ejection fraction. J Cardiovasc Magn Reson.

[21] Haaf P (2016). *C*ardiac T1 Mapping and Extracellular Volume (ECV) in clinical practice: a comprehensive review. J Cardiovasc Magn Reson.

[22] Brooks J, Kramer CM, Salerno M (2013). Markedly increased volume of distribution of gadolinium in cardiac amyloidosis demonstrated by T1 mapping. J Magn Reson Imaging.

[23] Flett, A. S. *et al*. Equilibrium contrast cardiovascular magnetic resonance for the measurement of diffuse myocardial fibrosis: preliminary validation in humans. *Circulation* **122**, 138–144, circulationaha.109.930636 [pii]10.1161/CIRCULATIONAH0A.109.930636 (2010). 10.1161/CIRCULATIONAHA.109.930636 20585010

[24] Lurz, J. A. *et al*. CMR-Derived Extracellular Volume Fraction as a Marker for Myocardial Fibrosis: The Importance of Coexisting Myocardial Inflammation. JAC*C Cardiovasc Imaging*, 10.1016/j.jcmg.2017.01.025 (2017). 10.1016/j.jcmg.2017.01.025 28412435

[25] Gheorghiade M, Filippatos G, De Luca L, Burnett J (2006). Congestion in acute heart failure syndromes: an essential target of evaluation and treatment. Am J Med.

[26] Hung, S. C., Lai, Y. S., Kuo, K. L. & Tarng, D. C. Volume overload and adverse outcomes in chronic kidney disease: clinical observational and animal studies. J Am *Heart Assoc* **4**, 10.1161/JAHA.115.001918 (2015). 10.1161/JAHA.115.001918 PMC4599419 25944876

[27] Wabel P, Chamney P, Moissl U, Jirka T (2009). Importance of whole-body bioimpedance spectroscopy for the management of fluid balance. Blood Purif.

[28] Rao NN, Dundon BK, Worthley MI, Faull RJ (2016). The Impact of Arteriovenous Fistulae for Hemodialysis on the Cardiovascular System. Semin Dial.

[29] Agarwal R (2003). Prevalence, treatment, and control of hypertension in chronic hemodialysis patients in the United States. Am J Med.

[30] Jofré R, Rodriguez-Benitez P, López-Gómez JM, Pérez-Garcia R (2006). Inflammatory syndrome in patients on hemodialysis. J Am Soc Nephrol.

[31] Korsheed S, Eldehni MT, John SG, Fluck RJ, McIntyre CW (2011). Effects of arteriovenous fistula formation on arterial stiffness and cardiovascular performance and function. Nephrol Dial Transplant.

[32] Iwashima Y (2002). Effects of the creation of arteriovenous fistula for hemodialysis on cardiac function and natriuretic peptide levels in CRF. Am J Kidney Dis.

[33] Stern AB, Klemmer PJ (2011). High-output heart failure secondary to arteriovenous fistula. Hemodial Int.

[34] Khreiss M (2009). High-output cardiac failure secondary to a large arteriovenous fistula: a persistent threat to the dialysis and kidney transplant patient. NDT Plus.

[35] Antlanger M (2013). Fluidoverload in hemodialysis patients: a cross-sectional study to determine its association with cardiac biomarkers and nutritional status. BMC Nephrol.

[36] Kalantar-Zadeh K, Block G, Humphreys MH, Kopple JD (2003). Reverse epidemiology of cardiovascular risk factors in maintenance dialysis patients. Kidney Int.

[37] Pfaffenberger S (2013). Siz*e* matters! Impact of age, sex, height, and weight on the normal heart size. Circ. Cardiovasc Imaging.

[38] Nagarajan V, Kohan L, Holland E, Keeley EC, Mazimba S (2016). Obesity paradox in heart failure: a heavy matter. ESC Heart Fail.

[39] Verbrugge FH (2017). Global myocardial oedema in advanced decompensated heart failure. Eur Heart J Cardiovasc Imaging.

[40] Dekker, M. J. *et al*. Impact of fluid status and inflammation and their interaction on survival: a study in an international hemodialysis patient cohort. Kidney *Int*, 10.1016/j.kint.2016.12.008 (2017). 10.1016/j.kint.2016.12.008 28209335

[41] Miller WL, Mullan BP (2016). Volume Overload Profiles in Patients With Preserved and Reduced Ejection Fraction Chronic Heart Failure: Are There Differences? A Pilot Study. JACC. Heart Fail.

