# Supplementary material for: Impact of Systemic Volume Status on Cardiac Magnetic Resonance T1 Mapping
Source: Sci Rep. 2018 Apr 3;8:5572. doi: 10.1038/s41598-018-23868-4 (PMC5882796; doi:10.1038/s41598-018-23868-4)

Supplementary Information for the manuscript

Impact of Systemic Volume Status on Cardiac Magnetic Resonance T1 Mapping

Marlies Antlanger, MD[1]; Stefan Aschauer, MD[2]; Andreas A. Kammerlander, MD[2]; Franz Duca, MD[2]; Marcus D. Säemann, MD[3,4]; Diana Bonderman, MD[2]; Julia Mascherbauer, MD[2]

[1] Department of Internal Medicine III, Division of Nephrology and Dialysis, Medical University of Vienna, Vienna, Austria.

[2] Department of Internal Medicine II, Division of Cardiology, Medical University of Vienna, Vienna, Austria.

[3] 6th Department of Internal Medicine, Nephrology and Dialysis, Wilhelminenspital, Vienna, Austria.

[4] Sigmund Freud Private University, Medical School, Vienna, Austria.

Supplementary Figure 1. Bar plots of native myocardial T1 time in healthy controls versus HD patients.

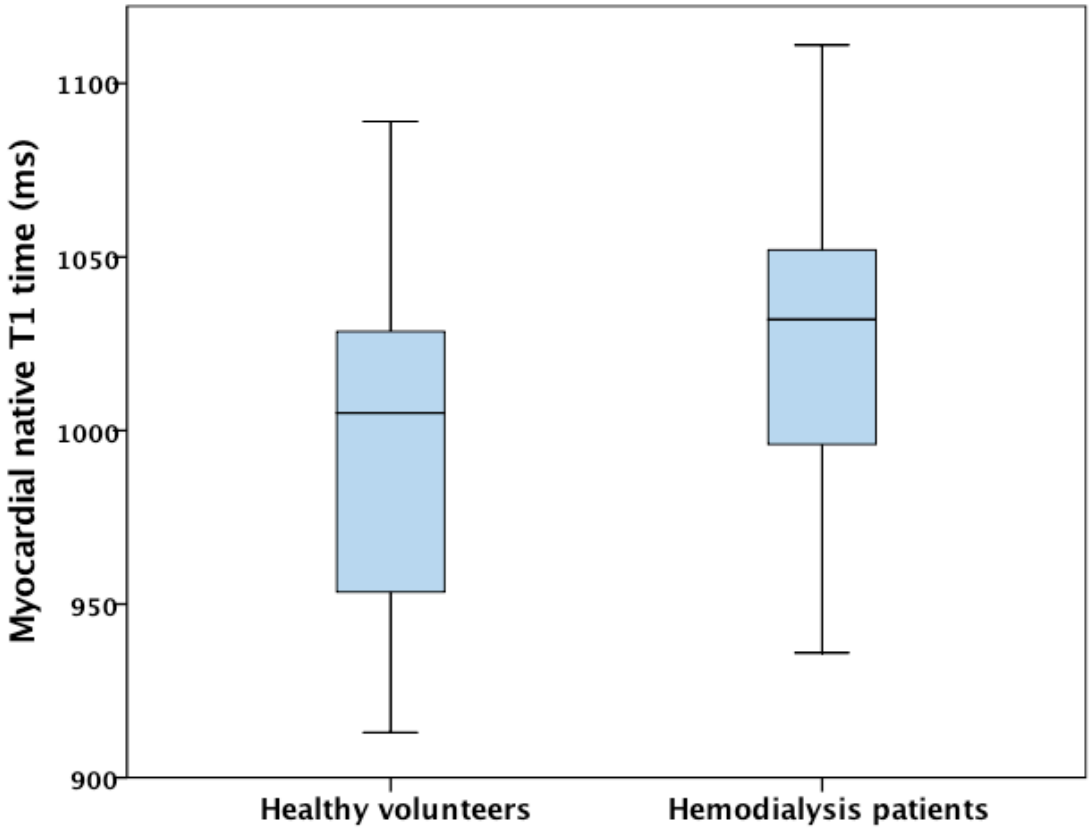

Supplementary Figure 2. Bar plots of native myocardial T1 time in normovolemic HD patients versus fluid overloaded HD patients versus healthy controls.

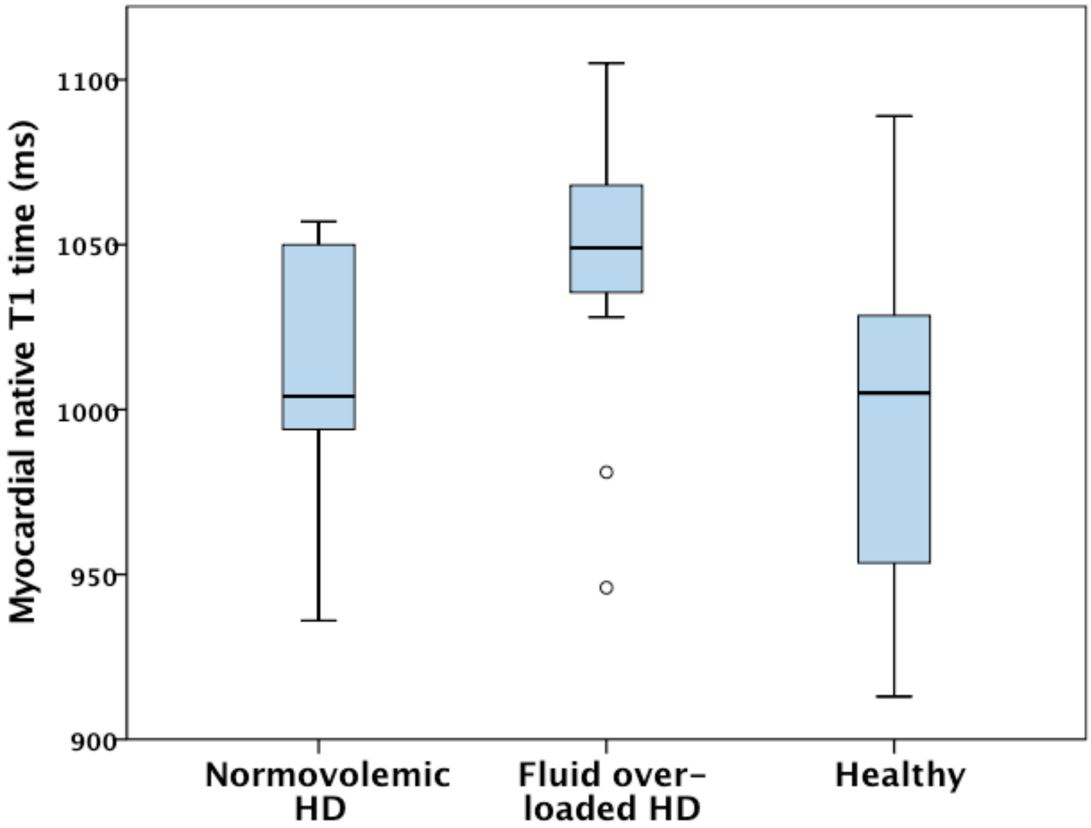

Supplementary Figure 3. Correlation plots of calculated post-HD fluid status and myocardial T1 time (short axis).

S3A. Absolute post-HD fluid status in Liters above/below ideal extracellular volume.

S3B. Relative post-HD fluid status in % above/below ideal extracellular volume.

Blue circles represent normovolemic patients; green circles represent fluid overloaded patients.

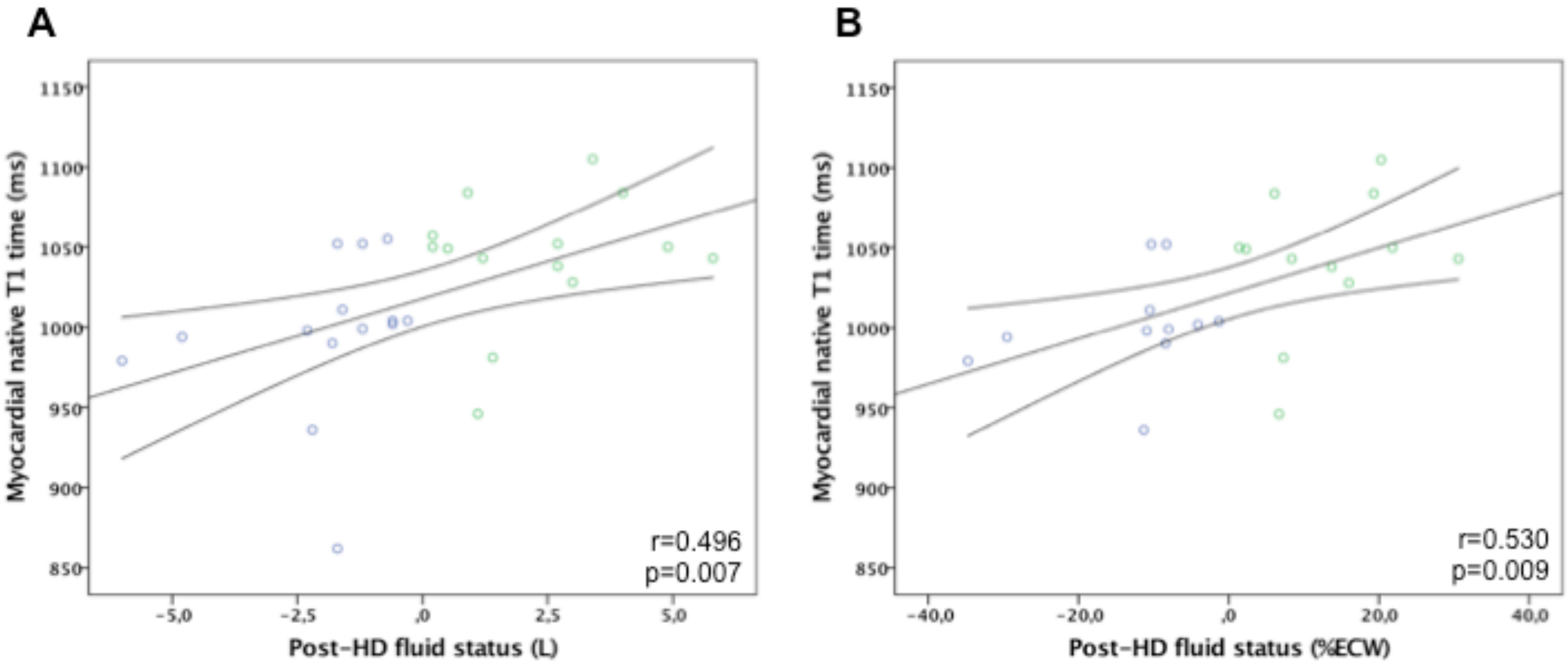

Supplement: Supplementary file 1 — Supplementary Figures [file 41598_2018_23868_MOESM1_ESM.pdf]
